# Supplementary material for: Relative and Absolute Quantification of Aberrant and Normal Splice Variants in HBBIVSI−110 (G > A) β-Thalassemia
Source: Int J Mol Sci. 2020 Sep 11;21(18):6671. doi: 10.3390/ijms21186671 (PMC7555009; doi:10.3390/ijms21186671)
Supplement: Supplementary file 1 [file ijms-21-06671-s001.pdf]

International Journal of *Molecular Sciences*

MDPI

Supplementary Figures

Relative and absolute quantification of aberrant and normal splice variants in $HBB^{IVSI\text{-}110(G>A)}$ β-thalassemia

Petros Patsali [1], Panayiota Papasavva [1,2], Soteroulla Christou [3], Maria Sitarou [4], Michael N. Antoniou [5], Carsten W. Lederer [1,2,†,*] and Marina Kleanthous [1,2,†]

1 Department of Molecular Genetics Thalassaemia, The Cyprus Institute of Neurology and Genetics, 1683 Nicosia, Cyprus; petrospa@cing.ac.cy (P.P.); panayiotap@cing.ac.cy (P.P.); marinakl@cing.ac.cy (M.K.)
2 Cyprus School of Molecular Medicine, 1683 Nicosia, Cyprus
3 Thalassaemia Clinic Nicosia, Ministry of Health, 1474 Nicosia, Cyprus; chrnchr@spidernet.com.cy
4 Thalassaemia Clinic Larnaca, Ministry of Health, 6301 Larnaca, Cyprus; msitarou@yahoo.gr
5 Department of Medical and Molecular Genetics, King's College London, London SE1 9RT, UK; michael.antoniou@kcl.ac.uk
* Correspondence: lederer@cing.ac.cy; Tel.: +357-22-392-764
† These authors have contributed equally to this study.

Abstract: The β-thalassemias are an increasing challenge to health systems worldwide, caused by absent or reduced β-globin (HBB) production. Of particular frequency in many Western countries is $HBB^{(IVSI-110)}$ β-thalassemia (HGVS name: HBB:c.93-21G > A). Its underlying mutation creates an abnormal splice acceptor site in the *HBB* gene, and while partially retaining normal splicing of *HBB*, it severely reduces HBB protein expression from the mutant locus and *HBB* loci in *trans*. For the assessment of the underlying mechanisms and of therapies targeting β-thalassemia, accurate quantification of aberrant and normal *HBB* mRNA is essential, but to date, has only been performed by approximate methods. To address this shortcoming, we have developed an accurate, duplex reverse-transcription quantitative PCR assay for the assessment of the ratio and absolute quantities of normal and aberrant mRNA species as a tool for basic and translational research of $HBB^{(IVSI-110)}$ β-thalassemia. The method was employed here to determine mRNA ratios and quantities in blood and primary cell culture samples and correlate them with HBB protein levels. Moreover, with its immediate utility for β-thalassemia and the mutation in hand, the approach can readily be adopted for analysis of alternative splicing or for quantitative assays of any disease-causing mutation that interferes with normal splicing.

Keywords: β-thalassemia; splice defect; duplex quantitative PCR; absolute quantification; transcript variants; splicing

---

Supplementary Figures

S1. Comparison of PCR kit performance in duplex reactions.

S2. Comparison of standard vs. half-volume RT-qPCR analyses.

S3. Alternative assessments of transcript quantities.

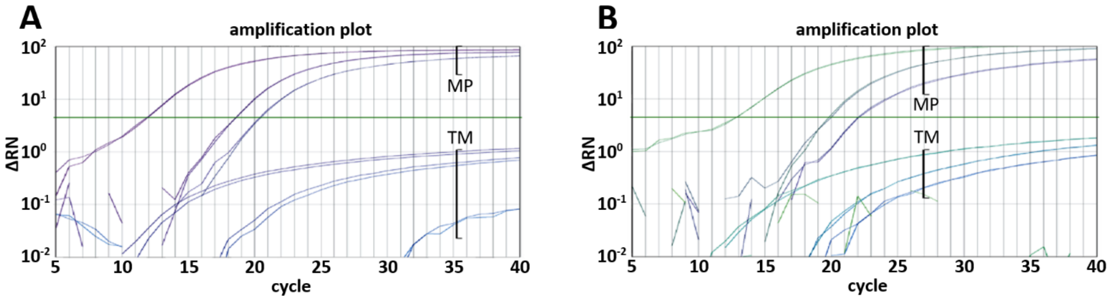

Supplementary Figure S1. Comparison of PCR kit performance in duplex reactions. Three equivalent dilutions of (**A**) normal template and (**B**) aberrant template were detected in duplex reactions containing both probes and amplicons using either the Multiplex PCR Kit (Qiagen, "MP") or the TaqMan Master Mix (Applied Biosystems, "TM"). Both amplification plots show the horizontal detection baseline {4.5} in green.

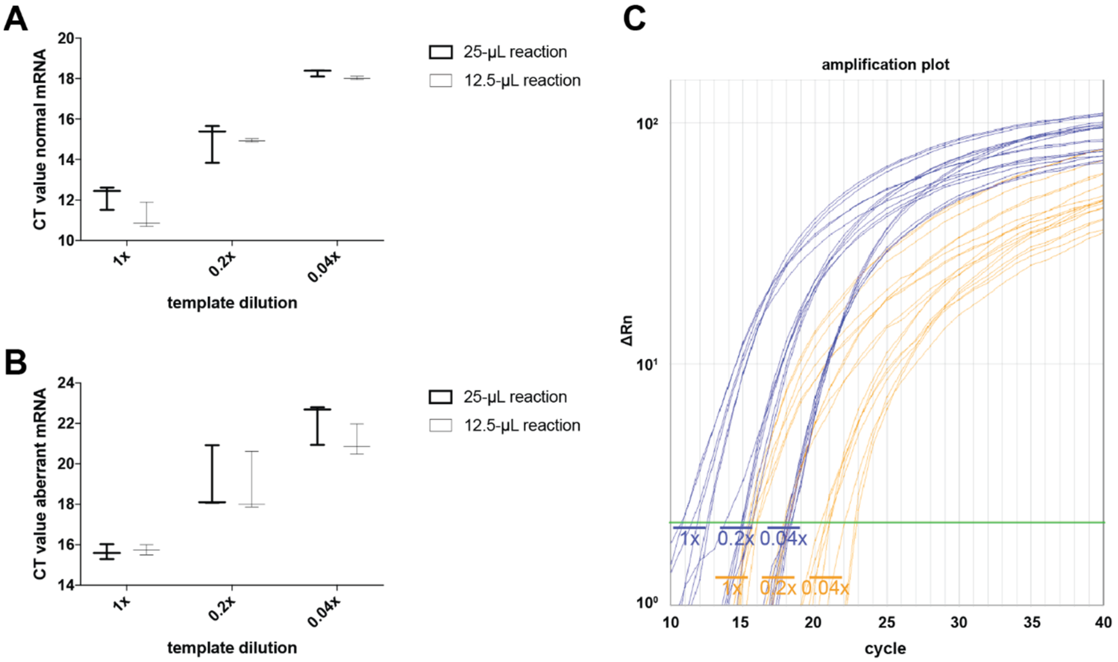

Supplementary Figure S2. Comparison of standard vs. half-volume RT-qPCR analyses. (**A**) Measurements for normal template quantities (based on probe N_ZNA_FAM) and (**B**) measurements for aberrant template quantities (based on probe A_MGB_VIC) were compared for standard (25-µL) and half-volume (12.5-µL) qPCR across three different split-plasmid template dilutions. The box-and-whisker diagrams show all three data points for each combination of template dilution and probe. (**C**) The corresponding amplification plot shows the horizontal detection baseline {1.2} in green and amplification of the aberrant template in orange and that of the normal template in blue, with the right-most three lines for each template and probe combination representing the half-volume reactions.

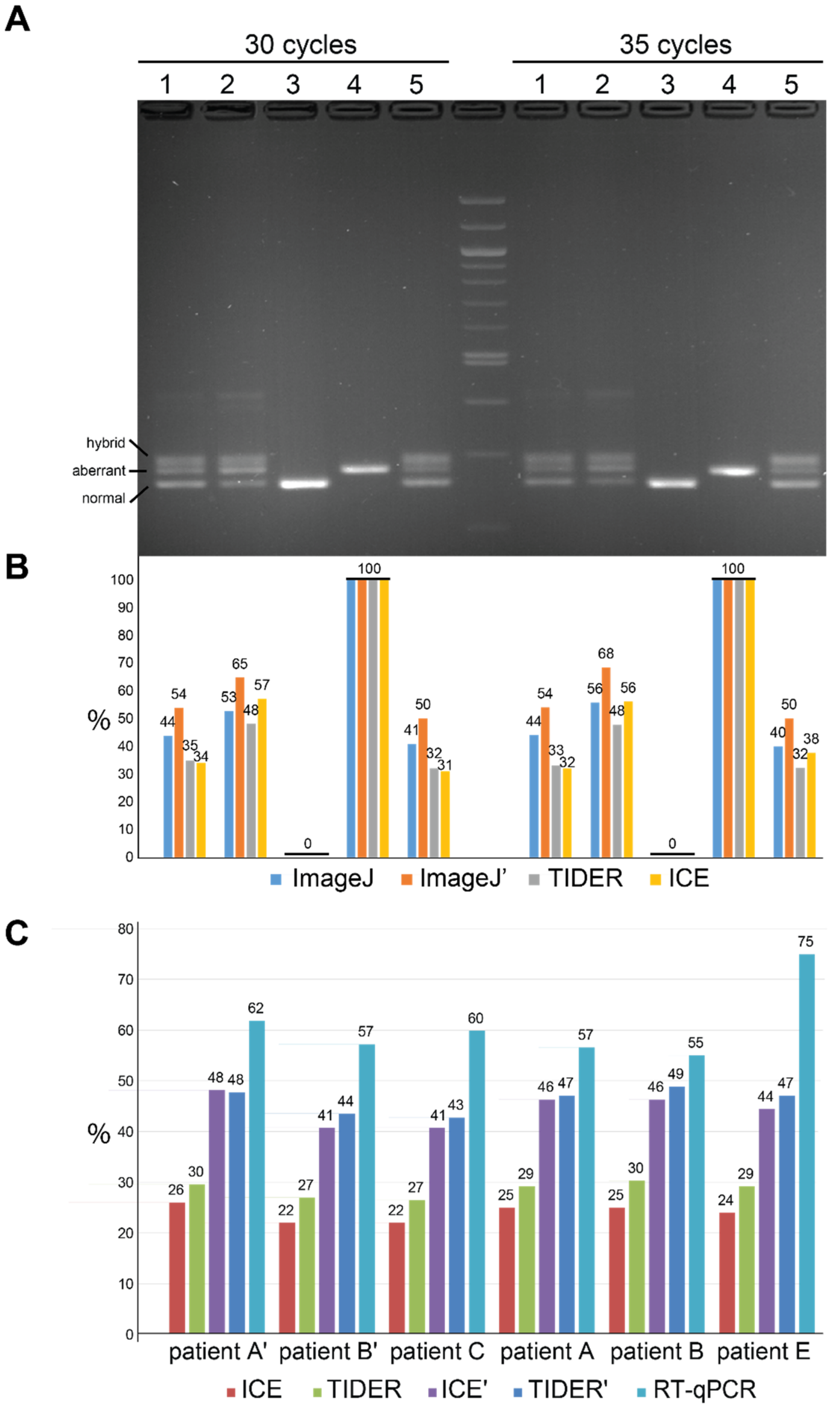

Supplementary Figure S3. Alternative assessments of transcript quantities. (**A**) Semi-quantitative PCR and end-point detection of amplicon bands representing aberrant, normal and hybrid PCR products for unrelated control samples, as indicated. Samples used were 300 ng purified PCR product each of (1) cDNA for patient A, (2) a pool of cDNA for multiple patient samples, (3) plasmid pCR2.1_HBB_N, (4) plasmid pCR2.1_HBB_A, (5) mixture 1:1 (based on spectrophotometric measurement) of pCR2.1_HBB_N and pCR2.1_HBB_A. (**B**) Comparison of ImageJ band quantification with decomposition of Sanger sequencing traces after cycle sequencing for samples shown in panel A and aligned with the corresponding gel lanes. Band intensities i (ImageJ) and fixed length n of each amplicon (122 bp for the aberrant amplicon [a], 103 bp for the normal amplicon [n], an average 112.5 bp for the hybrid amplicon [h]) were used to calculate relative contribution of aberrant

and normal HBB transcripts, as follows: %age aberrant RNA=100×((i[a]/n[a])+(i[h]/(2×n[h]))/((i[a]/n[a])+(i[h]/n[h])+(i[n]/n[n])). The ImageJ label indicates raw percentage measurement, the ImageJ′ label indicates percentage measurements rescaled for all samples in accordance with sample 5 holding 50% aberrant template. Based on the same PCR samples, TIDER and ICE HDR algorithms were used to decompose mixed sequence traces and estimate the relative abundance of aberrant HBB RNA based on end-point PCR. Across all samples, measurements correlate extremely well (Pearson $r \geq 0.959$, $p < 1.2 \times 10^{-5}$ for any two of the three measurement methods), excluding single-plasmid samples 3 and 4, measurements correlated well (Pearson $r \geq 0.918$, $p \leq 0.01$ for any two of the three measurement methods). However, ICE and TIDER in line with uncorrected ImageJ-derived quantities appear to underestimate the contribution of aberrant RNA. (**C**) Analysis of patient samples employed in this study by PCR (30 cycles), cycle sequencing and ICE- and TIDER-based decomposition of mixed sequence traces. Alignment of decomposition results with (ICE′, TIDER′) and without (ICE, TIDER) rescaling according to control samples holding 50% of aberrant template, and alignment with results obtained by RT-qPCR based quantification with fragmented (2i) standard curve design once more indicate a general underestimation of aberrant *HBB* RNA abundance by end-point PCR-based methods. There was no correlation with RT-qPCR based measurements (Pearson $r = 0.019$ and 0.089, for correlation with ICE and TIDER, respectively). For panels B and C, Y-axis labels and labels above individual columns indicate the relative quantity of aberrant *HBB*-derived transcripts as a percentage of all *HBB*-derived transcripts.

© 2020 by the authors. Submitted for possible open access publication under the terms and conditions of the Creative Commons Attribution (CC BY) license (http://creativecommons.org/licenses/by/4.0/).
